# KA‐mediated excitotoxicity induces neuronal ferroptosis through activation of ferritinophagy

**DOI:** 10.1111/cns.70054

**Published:** 2024-09-22

**Authors:** Yi‐Yue Jiang, Wei‐Long Wu, Jia‐Ni Huang, Na Liu, Jing Wang, Xiao‐Rui Wan, Zheng‐Hong Qin, Yan Wang

**Affiliations:** ^1^ Department of Pharmacology College of Pharmaceutical Sciences, Suzhou Key Laboratory of Aging and Nervous Diseases, and Jiangsu Key Laboratory of Neuropsychiatric Diseases Soochow University Suzhou China

**Keywords:** excitotoxicity, ferritinophagy, ferroptosis, NCOA4, neurodegenerative diseases

## Abstract

**Objectives:**

This study aims to elucidate the role of Fe^2+^ overload in kainic acid (KA)‐induced excitotoxicity, investigate the involvement of ferritinophagy selective cargo receptor NCOA4 in the pathogenesis of excitotoxicity.

**Methods:**

Western blotting was used to detect the expression of FTH1, NCOA4, Lamp2, TfR, FPN, and DMT1 after KA stereotaxic injection into the unilateral striatum of mice. Colocalization of Fe^2+^ with lysosomes in KA‐treated primary cortical neurons was observed by using confocal microscopy. Desferrioxamine (DFO) was added to chelate free iron, a CCK8 kit was used to measure cell viability, and the Fe^2+^ levels were detected by FerroOrange. BODIPY C11 was used to determine intracellular lipid reactive oxygen species (ROS) levels, and the mRNA levels of PTGS2, a biomarker of ferroptosis, were measured by fluorescent quantitative PCR. 3‐Methyladenine (3‐MA) was employed to inhibit KA‐induced activation of autophagy, and changes in ferritinophagy‐related protein expression and the indicated biomarkers of ferroptosis were detected. Endogenous NCOA4 was knocked down by lentivirus transfection, and cell viability and intracellular Fe^2+^ levels were observed after KA treatment.

**Results:**

Western blot results showed that the expression of NCOA4, DMT1, and Lamp2 was significantly upregulated, while FTH1 was downregulated, but there were no significant changes in TfR and FPN. The fluorescence results indicated that KA enhanced the colocalization of free Fe^2+^ with lysosomes in neurons. DFO intervention could effectively rescue cell damage, reduce intracellular lipid peroxidation, and decrease the increased transcript levels of PTGS2 caused by KA. Pretreatment with 3‐MA effectively reversed KA‐induced ferritinophagy and ferroptosis. Endogenous interference with NCOA4 significantly improved cell viability and reduced intracellular free Fe^2+^ levels in KA‐treated cells.

**Conclusion:**

KA‐induced excitotoxicity activates ferritinophagy, and targeting ferritinophagy effectively inhibits downstream ferroptosis. Interference with NCOA4 effectively attenuates KA‐induced neuronal damage. This study provides a potential therapeutic target for excitotoxicity related disease conditions.

## Graphical abstract

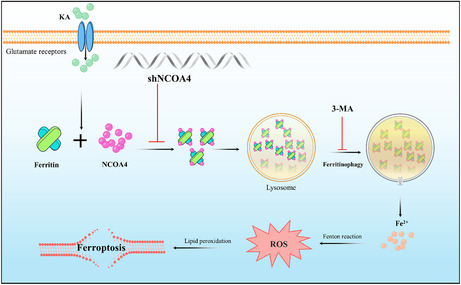

## 1 INTRODUCTION

Neurodegenerative disease is a major health problem characterized by the loss and progressive degeneration of neurons in the central nervous system (CNS). Due to the improvement of living environment and the extension of life expectancy, the incidence of neurodegenerative diseases has greatly increased worldwide.^1^, ^2^ Glutamate is the main excitatory neurotransmitter in the CNS of mammals,^3^ and excessive stimulation of glutamate increases intracellular Ca^2+^ levels by directly opening postsynaptic ion channels and indirectly affecting calcium homeostasis mechanisms.^4^, ^5^ Neurodegenerative diseases associated with glutamate dysfunction share common pathogenic mechanisms.^4^ According to reports, excitotoxicity is associated with cellular damage and death mechanisms such as autophagy, oxidative stress, and neuroinflammation.^6^, ^7^, ^8^, ^9^ However, the underlying mechanisms of excitotoxicity require further exploration.

Ferroptosis is a recently discovered form of programmed cell death characterized by the accumulation of reactive oxygen species (ROS) and lipid peroxides within the cell.^10^, ^11^ Ferroptosis has distinct biochemical, morphological, and genetic differences from other cell death mechanisms, such as autophagy, apoptosis, and necrosis. The primary morphological changes in cells undergoing ferroptosis include mitochondrial membrane condensation, mitochondrial crista disruption, and outer membrane rupture or disappearance.^12^ In addition, this type of cell death is characterized by iron deposition and lipid peroxidation, as well as changes in key genes or proteins involved in lipid metabolism, such as acyl‐CoA synthetase long‐chain family member 4 (ACSL4) and prostaglandin‐endoperoxide synthase 2 (PTGS2).^13^ Although the typical phenomenon of ferroptosis has been observed in various neurodegenerative diseases, the specific mechanism and regulatory targets are still unclear. Overactivation of the glutamate receptor has been found to not only cause Ca^2+^ influx but also induce N‐methyl‐D‐aspartic acid (NMDA) receptor‐mediated iron uptake.^14^ Ferroptosis inhibitors effectively counteract glutamate‐induced hippocampal neurotoxicity, indicating that lipid ROS‐induced cell death and ferroptosis play important roles in neuronal excitotoxicity.^10^

In addition to the metabolic regulation of iron entering and exiting cells, numerous studies suggest that overactivation of autophagy and lysosomes can degrade ferritin, increase unstable iron accumulation, and promote iron‐dependent cell death.^15^ Ferritin is composed of ferritin heavy chain 1 (FTH1) and ferritin light chain (FTL) and is primarily responsible for intracellular iron storage, with the ability to chelate up to 4500 Fe^3+^ ions.^16^ Initial studies found that in the case of iron depletion, ferritin is transported to lysosomes and degraded through the autophagy pathway.^17^ However, in 2014, nuclear receptor coactivator 4 (NCOA4) was identified by two independent research groups as a selective receptor for ferritinophagy, and it was found that NCOA4 directly binds to FTH1 and transports it into the lysosome.^18^, ^19^

Evidence shows that NCOA4 mRNA and protein are expressed in the brains of mice,^20^ but there is currently no research on the expression level or function of NCOA4 in pathological specimens of patients with aging or neurodegenerative diseases. Additionally, there are no known pathogenic mutations in NCOA4 associated with CNS diseases.^21^ Loss of NCOA4 reduces ferritin degradation, resulting in iron retention and decreased bioavailable iron in vitro.^22^ This reduction causes a decrease in oxidative stress levels and a corresponding decrease in sensitivity to iron‐dependent death, indicating that the inhibition of NCOA4 may have a protective effect on the brain in neurodegenerative diseases. The expression and function of NCOA4 in the brain and its role in neurodegenerative disease are unexplored areas of research, and it is necessary to further study its molecular mechanisms and signaling pathways.

In this study, we explored the main mechanisms of free iron overload in kainic acid (KA)‐induced excitotoxicity and investigated the potential effect of ferritinophagy on KA‐induced ferroptosis.

## 2 MATERIALS AND METHODS

### 2.1 Animal treatment

Male ICR mice (6–8 weeks, 20–25 g) were purchased from Suzhou Zhaoyan New Drug Research Center. They were maintained under controlled temperature (22°C) and humidity (40%–70%) conditions with good ventilation and a 12 h/12 h light/dark cycle. Mice had access to unlimited water and food. All animal experiments were approved by the Institutional Animal Care and Use Committee of Soochow University.

We prepared a KA solution at a concentration of 0.625 nmol/μL in normal saline. After anesthetizing the mice, 1 μL of the KA solution was injected into the right striatum of each mouse, while the control group received an injection of normal saline at the same location. The injection coordinates were as follows: 0.8 mm anterior to bregma, 1.8 mm lateral to the sagittal suture, and 3.5 mm ventral to the pial surface. The needle was left in place for 5 min after injecting the solution within 2 min. The method of constructing an in vivo excitotoxicity model was as described previously.^23^

Mice were injected with 15 μg per individual of 3‐MA in the right striatum 30 min prior to KA injection. The administration method of 3‐MA was the same as described previously.^7^

### 2.2 Cell culture and treatment

The perforated plate or dish was encapsulated with polycation poly‐L‐lysine. The cerebral cortex was separated from 16‐ to 18‐day‐old fetal mice, and the meninges were removed. Forceps were used to divide the cortex into approximately 1 mm^3^ tissue fragments. Then, 0.25% trypsin was added, the cells were placed in a 37°C incubator for 15 min, and 10% complete medium was added to terminate the digestion. Fifty microliters of DNase I was added and gently pipetted until the tissue was disrupted into a single‐cell suspension and centrifuged at 1500 × *g* for 5 min. The supernatant was discarded, neurobasal medium was added to resuspend the cells, the precipitate was completely dispersed, and the cells were filtered through a 40 μm cell strainer to obtain a single cell suspension. After counting, the cell suspension was diluted to 1 × 10^6^ cells/mL and seeded in a pre‐encapsulated plate. The primary neurons could be used for experiments after 7–8 days of culture.

The HT22 cell line of hippocampal neurons in mice was purchased from Fuheng Biology. The cells were cultured in Dulbecco's modified Eagle medium (DMEM) containing 10% fetal bovine serum (FBS) and 1% (v/v) penicillin–streptomycin at 37°C in a 5% CO_2_ incubator.

Primary neurons were cotreated with DFO (Sigma Aldrich, D9533) and KA for 8 h or pretreated with 3‐MA (MCE, HY‐19312) for 4 h before KA treatment.

### 2.3 Cell viability assay

We assessed cell viability using the Cell Counting Kit‐8 (CCK8). After drug treatment, a CCK8 working solution was prepared and added to a 96‐well plate. The plate was then incubated at 37°C and 5% CO_2_ for an appropriate time, and the absorbance was measured at 450 nm.

### 2.4 Small hairpin RNA (shRNA) and transfection

The RNAi sequence targeting NCOA4 was designed as CTCCTCAAGTATTGGGCCTTT, and negative control vectors were synthesized and packaged by Genechem Co., Ltd. HT22 cells were seeded at a density of 5 × 10^4^ cells/well in a 24‐well plate and transfected with NCOA4‐targeting shRNA for 12 h in medium, followed by selection of stable transfectants using 1 μg/μL puromycin. Total cell proteins and RNA were extracted for western blot and RT‐qPCR.

### 2.5 Nissl staining

In this study, cardiac perfusion was conducted on the mice to obtain their brains. Brain coronal sections measuring 40 μm were prepared through a gradient dehydration method using 20% and 30% sucrose solutions. The sections were then subjected to Nissl staining solution for 30 min, followed by a gradient decolorization process using 75%, 95%, and absolute ethanol. Finally, the sections were rendered transparent with xylene. Observations were made using a microscope at 20× magnification to facilitate counting. Nissl staining and quantification were performed as previously described.^8^

### 2.6 Lipid peroxidation analysis

When the unsaturated butadiene group in BODIPY 581/591 C11 undergoes oxidation–reduction reactions with reactive oxygen species in the cell membrane, the fluorescence emission peak shifts from 590 nm to 510 nm.^24^ In this study, after primary cortical neuron treatment, we subsequently removed them from the culture medium. The samples were washed with phosphate‐buffered saline (PBS) and then treated with 5 μM BODIPY 581/591 C11 working solution (Invitrogen, D3861). Following a 30 min reaction period, the cells were incubated at 37°C with 5% CO_2_ in a culture incubator. The images were captured using a 60× objective lens on a laser confocal microscope and analyzed with ZEN software.

After incubation with the BODIPY 581/591 C11 probe, we also digested and collected cells using 0.1% trypsin and analyzed lipid peroxidation levels using flow cytometry (BD Bioscience, BD ARAШ).

### 2.7 Measurement and localization of intracellular Fe^2+^

Cells were seeded in a 24‐well plate containing small round glass slides. Upon completion of drug treatment, the culture medium was discarded. FerroOrange (Dojindo, F374) working solution (1 μmol/L) was added to each well. The reaction proceeded for 45 min at 37°C and 5% CO_2_ in a cell culture incubator. Hoechst 33342 working solution (1 μg/mL) was then added to stain the nuclei for 15 min. Finally, images were collected using a fluorescence microscope.

Cells were sequentially incubated with FerroOrange and Lyso‐tracker (Beyotime, C1047S) working solutions, and the localization of Fe^2+^ in lysosomes was observed under a confocal microscope (Carl Zeiss, LSM 710).

### 2.8 Western blot analysis

Mouse tissues and cell samples were lysed with RIPA buffer, briefly sonicated, and then centrifuged at 12500 rpm for 20 min to obtain the supernatant as a protein sample. The protein concentration was determined by the BCA method (Beyotime, P0012). Western blotting was conducted as described previously.^25^ The following primary antibodies were used: anti‐GAPDH (Abcam, ab37168), anti‐β‐actin (Sigma Aldrich, A5441), anti‐TfR (Novus, NB200‐585ss), anti‐FPN (Abcam, ab78066), anti‐DMT1 (Abmart, ps‐3577), anti‐FTH1 (ABclonal, A19544), and anti‐NCOA4 (ABclonal, A5694).

### 2.9 Quantitative real‐time PCR (RT‐qPCR) analysis

According to the manufacturer's protocol, TRIzol was used to extract RNA from primary cortical neurons, and 1 μg of RNA was reverse transcribed into cDNA using RT SuperMix for qPCR (Vazyme, R323‐01). QPCR results were indicated by SYBR qPCR Master Mix (Vazyme, Q711‐02) with a final volume of 20 μL. Polymerase chain reaction conditions were as follows: predenaturation (95°C for 30 s), amplification and quantification program repeated for 40 cycles (95°C for 10 s, 60°C for 15 s), and melt curve analysis. The primers were designed and synthesized by Shanghai Sangon Biotech. The expression level of the target gene was normalized to that of GAPDH.

GAPDH‐Forward: TGGAGAAACCTGCCAAGTATG.

GAPDH‐Reverse: CCTGTTGCTGTAGCCGTATTC.

PTGS2‐Forward: TTCCAATCCATGTCAAAACCGT.

PTGS2‐Reverse: AGTCCGGGTACAGTCACACTT.

NCOA4‐Forward: CCTGGGGCAATCTGAAGGG.

NCOA4‐Reverse: CTGAGGAGTCACCAACCAATC.

### 2.10 Statistical analysis

Statistical analysis was performed using GraphPad Prism version 8.0. All data are expressed as the mean ± SEM. Prior to all data analysis, the Shapiro–Wilk test was used to test the normal distribution of the data. If the data are normally distributed with uniform variance, unpaired *t* tests were used to analyze the differences between two groups, and the differences among multiple groups were analyzed by one‐way ANOVA with Tukey's multiple comparison test. Otherwise, data were analyzed using the Kruskal–Wallis test. *p < 0.05* was considered statistically significant.

## 3 RESULTS

### 3.1 KA‐mediated excitotoxicity induced neuronal damage and ferritinophagy

**FIGURE 1:**
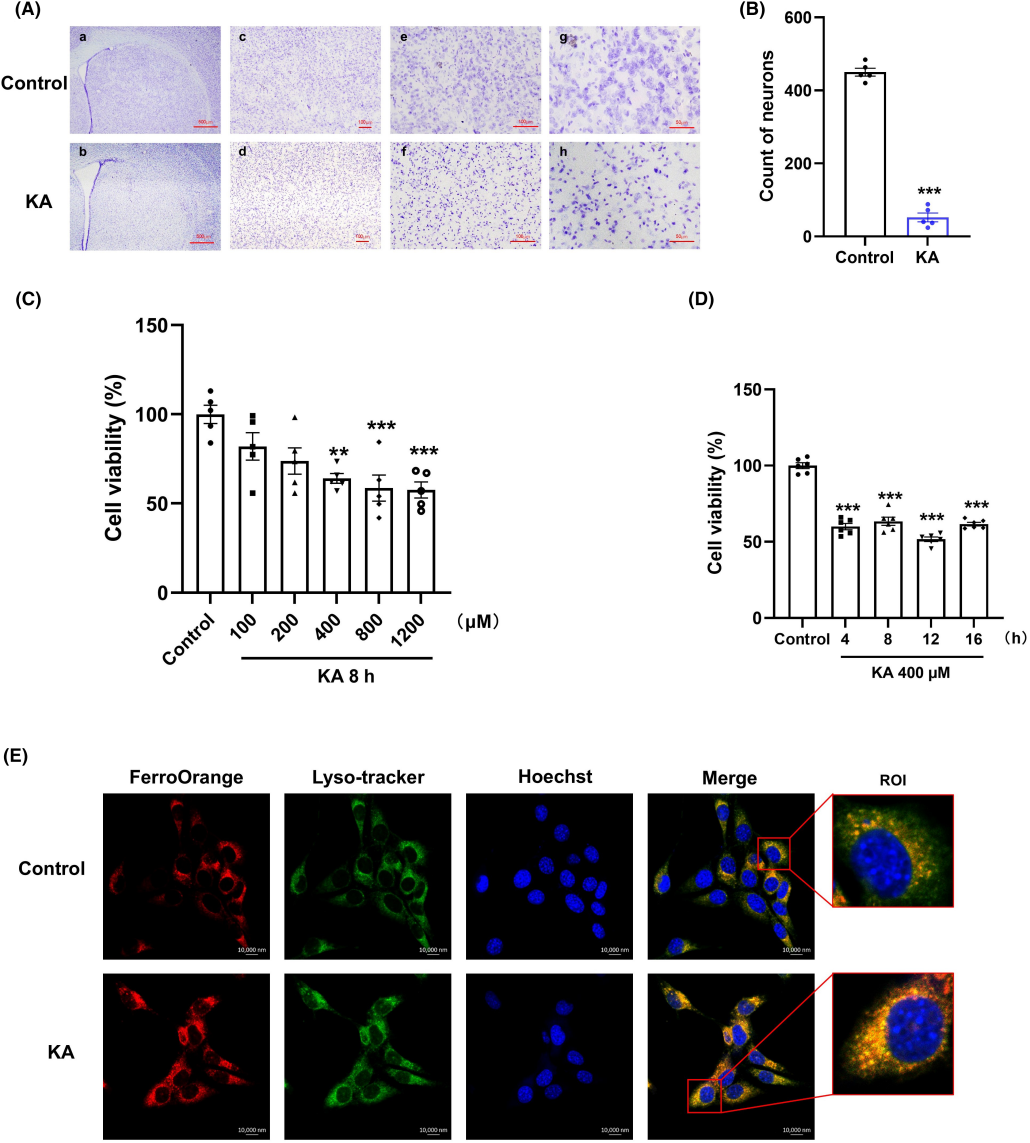
Neuronal damage and ferritinophagy induced by KA‐mediated excitotoxicity. (A) Mouse brain sections were obtained 14 days after 0.625 nmol KA was stereotaxically injected into the unilateral striatum. Representative brain sections of the striatum with Nissl staining are shown. Scale bar = 500 μm in a,b; 200 μm in c,d; 100 μm in e,f; and 50 μm in g,h. (B) Quantitative statistics of morphologically functioning neurons (*n* = 5). (C) Dose response of KA‐induced cytotoxicity. HT22 cells were treated with specified concentrations (100, 200, 400, 800, and 1200 μM) of KA for 8 h, and cell viability was determined using a CCK8 assay (*n* = 5). (D) Time course of KA‐induced cytotoxicity. HT22 cells were treated with 400 μM KA for 4, 8, 12, and 16 h, and cell viability was determined using a CCK‐8 assay (*n* = 6). (E) Colocalization image of FerroOrange (red) and Lyso‐tracker (green) in HT22 cells treated with 400 μM KA for 4 h (scale bar = 10 μm). Data are expressed as the mean ± SEM. **p < 0.01 vs. control, ^***^ *p* < 0.001 vs. control.

In this study, in vivo and in vitro excitotoxicity models were constructed using a KA receptor agonist. After injecting KA into the unilateral striatum of mice for 14 days, coronal brain slices were prepared to observe the appearance of Nissl bodies, which are markers of neuronal functional status. Following KA administration, a significant decrease in the number of Nissl bodies, morphological shrinkage and increased staining intensity were observed in the striatum (Figure 1A,B). Then, different concentrations of KA were administered to HT22 cells for 8 h to determine cell viability. It was found that 400 μM KA could significantly damage HT22 cells (Figure 1C). Based on this, we sought the optimum time for KA injury, and 40% of the cells died after being treated with KA for 4 h (Figure 1D). These results indicated that KA caused neuronal death both in vitro and in vivo. Therefore, we selected 400 μM KA and 4 h of treatment. At the same time, the use of specific probes revealed a significant increase in the colocalization of free Fe^2+^ and lysosomes (Figure 1E), indicating that KA could also activate ferritinophagy in HT22 cells.

### 3.2 Ferritinophagy rather than iron transmembrane transport is involved in KA‐induced ferroptosis

**FIGURE 2:**
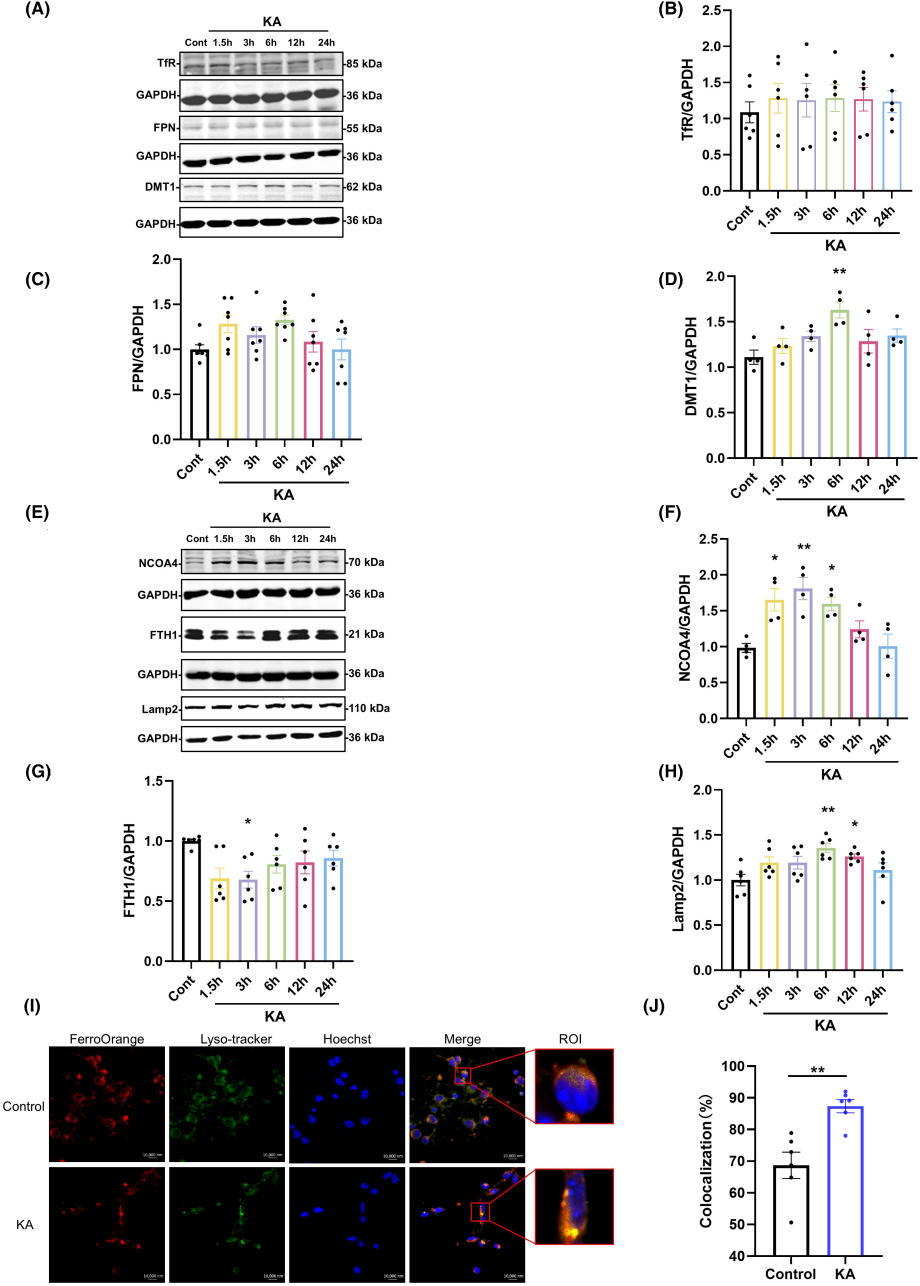
Ferritinophagy rather than iron transmembrane transport is involved in KA‐induced ferroptosis. (A–D) Representative bands and semiquantitation of western blots for detecting TfR, FPN, and DMT1 protein levels. (E–H) Representative bands and semiquantitation of western blots for detecting NCOA4, FTH1, and Lamp2 protein levels. (I,J) Confocal images and colocalization statistics of FerroOrange (red) and Lyso‐tracker (green) after 8 h of treatment of primary cortical neurons with KA (100 μM) (scale bar = 10 μm); the region of interest (ROI) is an enlarged image of a single cell (*n* = 6). Data are expressed as the mean ± SEM. *p < 0.05 vs. control, **p < 0.01 vs. control.

In the excitotoxicity mediated by KA, free iron overload is a key step in causing iron‐dependent neuronal death. Based on previous findings by our research group that unilateral striatal injection of KA results in elevated levels of total iron and Fe^2+^ in brain tissue, the specific mechanism by which KA increases free iron overload remains to be elucidated. The intracellular concentration of iron ions is mainly regulated by proteins such as TfR, FPN, DMT1, and FTH1.^26^ Among these, TfR and FPN are the key proteins for Fe^2+^ to enter and exit cells, and DMT1 is essential for the transport of divalent metal ions into cells. In this study, changes in iron transport‐related proteins were detected by western blot at 0, 1.5, 3, 6, 12, and 24 h after unilateral striatal injection of KA in mice. The results showed that there were no significant changes in TfR and FPN levels within 24 h, while DMT1 levels showed a transient increase at 6 h (Figure 2A–D). The above results indicate that there is a disturbance in intracellular Fe^2+^ metabolism during the early stages of excitotoxicity, while free iron overload may not be related to transmembrane transport of iron ions.

In the process of iron metabolism in neurons, in addition to the transmembrane transport of iron, another process related to the concentration of free iron is the activity of ferritin. Under some pathological conditions, FTH1 is specifically recognized by NCOA4, transported to lysosomes for degradation, and then released as free iron.^27^ To verify whether ferritinophagy occurs, changes in the protein expression of FTH1, NCOA4, and Lamp2, proteins related to autophagy, were determined in the striatum of mice injected with KA. As shown in the figure, FTH1 levels decreased at 3 h, and correspondingly, NCOA4 was upregulated at 1.5–6 h, with the most significant upregulation at 3 h; Lamp2 is a component of lysosomal membranes and a marker of lysosomes, and the upregulation of Lamp2 at 6–12 h indicates a significant activation response of lysosomes (Figure 2E–H).

During the process of ferritinophagy, FTH1 significantly accumulates in lysosomes and releases Fe^2+^ into the cytoplasmic LIP. To further assess the occurrence of ferritinophagy and subcellular processes, confocal microscopy was used to observe the colocalization of Fe^2+^ and lysosomes in primary cortical neurons. Fluorescent imaging demonstrated that in neurons treated with KA, the colocalization of Fe^2+^ and lysosomes significantly increased (Figure 2I,J). This finding indicated that during excitotoxicity, more Fe^2+^ was released from lysosomes. These results show that KA may induce the degradation of FTH1 through the lysosomal autophagy pathway.

### 3.3 Alleviation of iron overload protects primary neurons from KA‐induced ferroptosis

**FIGURE 3:**
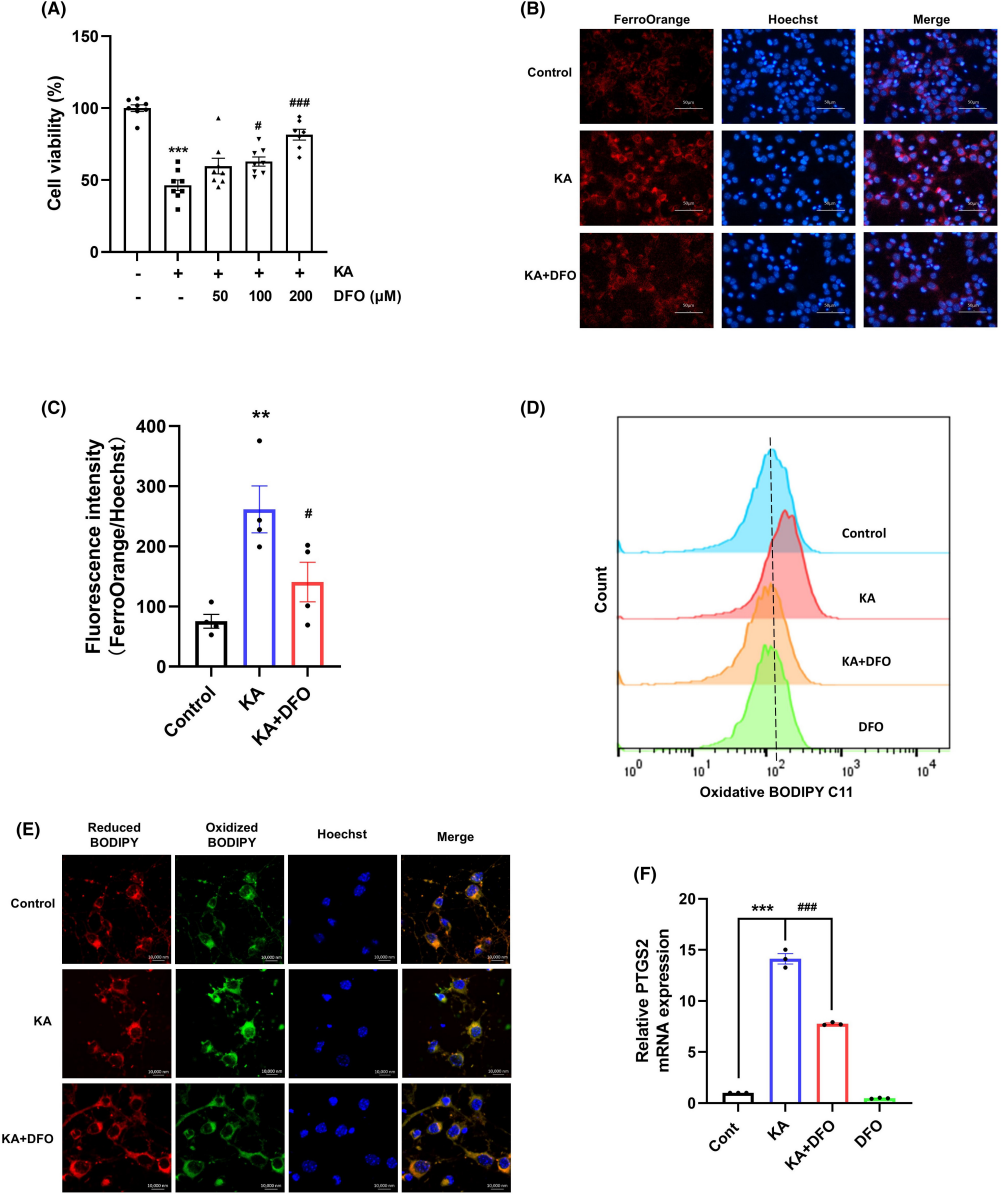
DFO alleviated KA‐induced neuronal ferroptosis. (A) Primary cortical neurons were treated with KA (100 μM) and different concentrations of DFO (50, 100, and 200 μM) for 8 h, and CCK8 was used to detect cell viability (*n* = 8). (B,C) Representative images and quantitative statistics of intracellular Fe^2+^ in primary neurons (scale bar = 50 μm) (*n* = 4). (D) Flow cytometric analysis of lipid ROS (*n* = 3). (E) Confocal image of reduced (red)/oxidized (green) BODIPY C11 in neurons and nuclei imaged using Hoechst 33342 (blue) (scale bar = 10 μm). (F) Real‐time fluorescence quantitative PCR detection of mRNA levels of PTGS2 (*n* = 3). Data are expressed as the mean ± SEM. **p < 0.01 vs. control, ***p < 0.001 vs. control, ^#^ *p* < 0.05 vs. KA‐treated group, ^###^ *p* < 0.001 vs. KA‐treated group.

We observed elevated total iron and ferrous ion levels in the KA‐injected striatum. To further determine the role of iron in the progression of excitotoxicity, we treated primary mouse cortical neurons with DFO as an iron chelator to decrease the intracellular iron concentration and exposed the cells to KA for 8 h. DFO treatment increased the number of surviving cells (Figure 3A). Intracellular ferrous ion levels increased significantly after KA treatment and were effectively inhibited by DFO treatment (Figure 3B,C). Furthermore, lipid peroxidation is a common event of ferroptosis, and PTGS2 expression level is one of the markers of ferroptosis. DFO reversed KA‐induced lipid peroxidation (Figure 3D,E) and KA‐induced increase of PTGS2 mRNA expression level (Figure 3F). Thus, the DFO‐mediated reduction in intracellular iron levels decreased ferroptosis and increased the survival of primary neurons.

### 3.4 Inhibition of autophagy ameliorated KA‐induced ferroptosis in primary neurons

**FIGURE 4:**
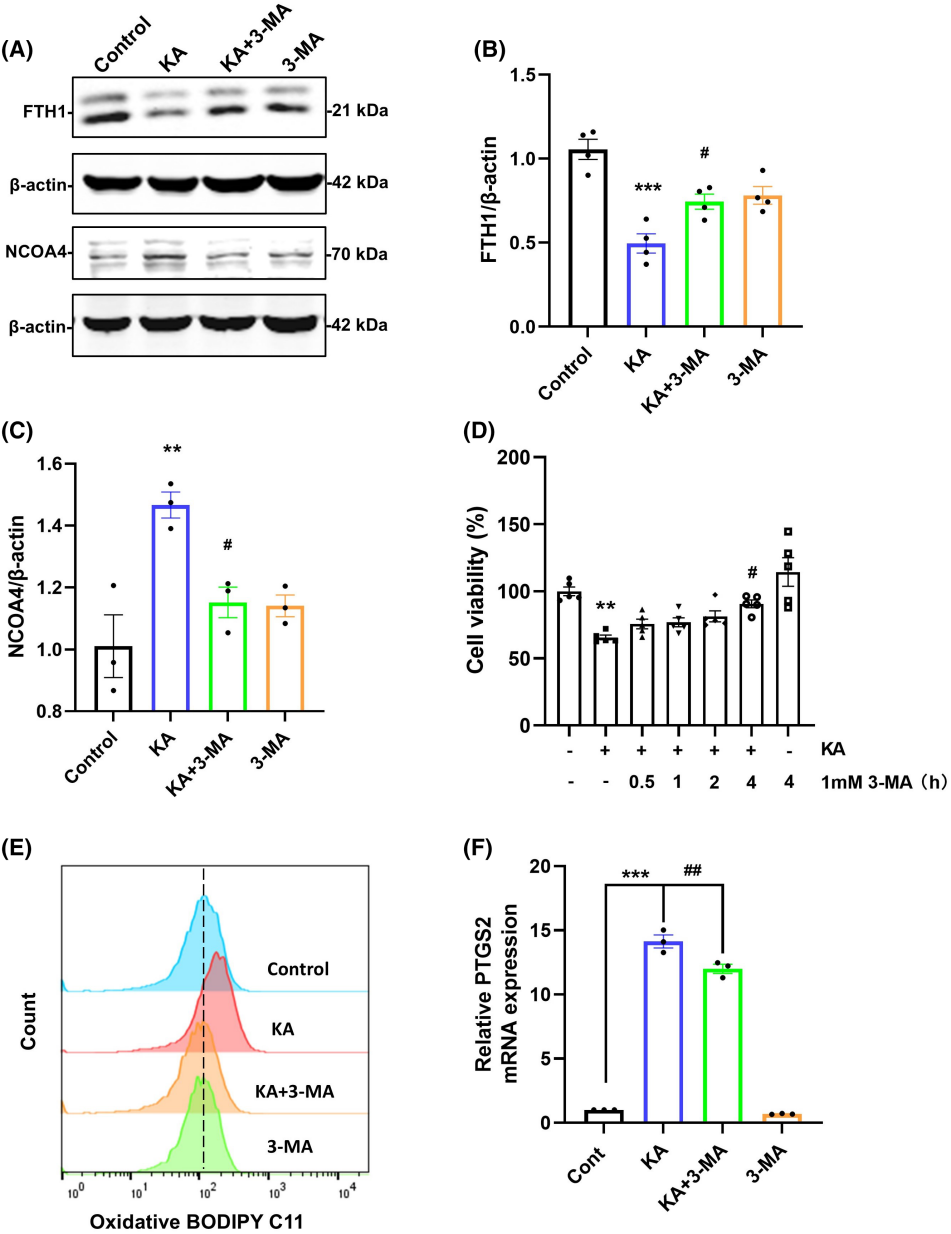
3‐MA inhibited KA‐induced neuronal ferroptosis. (A–C) Representative bands and semiquantitation of western blots for detecting NCOA4 and FTH1 protein levels. (D) Cell viability of primary neurons was assessed by pretreating the neurons with 3‐MA (1 mM) for 0.5, 1, 2, and 4 h, followed by treatment with KA (100 μM) for 8 h and determination by the CCK8 assay (*n* = 5). (E) Flow cytometric analysis of lipid ROS levels (*n* = 3). (F) Real‐time fluorescence quantitative PCR detection of mRNA levels of PTGS2 (*n* = 3). Data are expressed as the mean ± SEM. **p < 0.01 vs. control, ***p < 0.001 vs. control, ^#^ *p* < 0.05 vs. KA‐treated group, ^##^ *p* < 0.01 vs. KA‐treated group.

There is no clear evidence supporting the role of autophagy and ferroptosis in excitotoxic neurotoxicity, although early reports suggest that the mechanisms of ferroptosis are independent of those of other cell death modes. However, increasing evidence indicates that ferroptosis requires autophagy‐related processes, such as NCOA4‐mediated ferritinophagy, RAB7A (a member of the Ras oncogene family)‐mediated lipophagy, and Beclin1‐mediated inhibition of the x_c_^−^ system.^15^ Our group has previously identified autophagy in excitotoxicity,^7^, ^8^ and a recent study identified ferritinophagy as a distinct form of autophagy that mediates ferritin degradation and releases free iron.^18^ To confirm the involvement of ferritinophagy, we injected 3‐MA into the striatum 30 min before KA treatment. As shown in the results, FTH1 was downregulated after KA treatment but upregulated after 3‐MA pretreatment (Figure 4A,B), and 3‐MA also reversed the KA‐induced increase in NCOA4 levels (Figure 4A,C).

Based on the protective effect of 3‐MA inhibition on ferritin, we propose a hypothesis that inhibiting autophagy in excitotoxicity can reduce the occurrence of ferroptosis. To test this hypothesis, we pretreated primary neurons with 1 mM 3‐MA for different times before KA injury, and the CCK8 results showed that 3‐MA pretreatment for 4 h exhibited a suitable protective effect against injury, increasing the cell survival rate by 17% (Figure 4D). We then tested lipid ROS levels and the transcription levels of PTGS2, and flow cytometry results showed that 3‐MA pretreatment reduced the oxidative‐type BODIPY C11 peak shift caused by KA and effectively reduced the abnormally elevated PTGS2 mRNA levels (Figure 4E,F). These results indicate that autophagy inhibition effectively alleviates the increase in ferroptosis indicators caused by KA, indicating that autophagy is involved in KA‐induced ferroptosis.

### 3.5 NCOA4 regulated ferritinophagy mediates KA induced excitotoxicity

**FIGURE 5:**
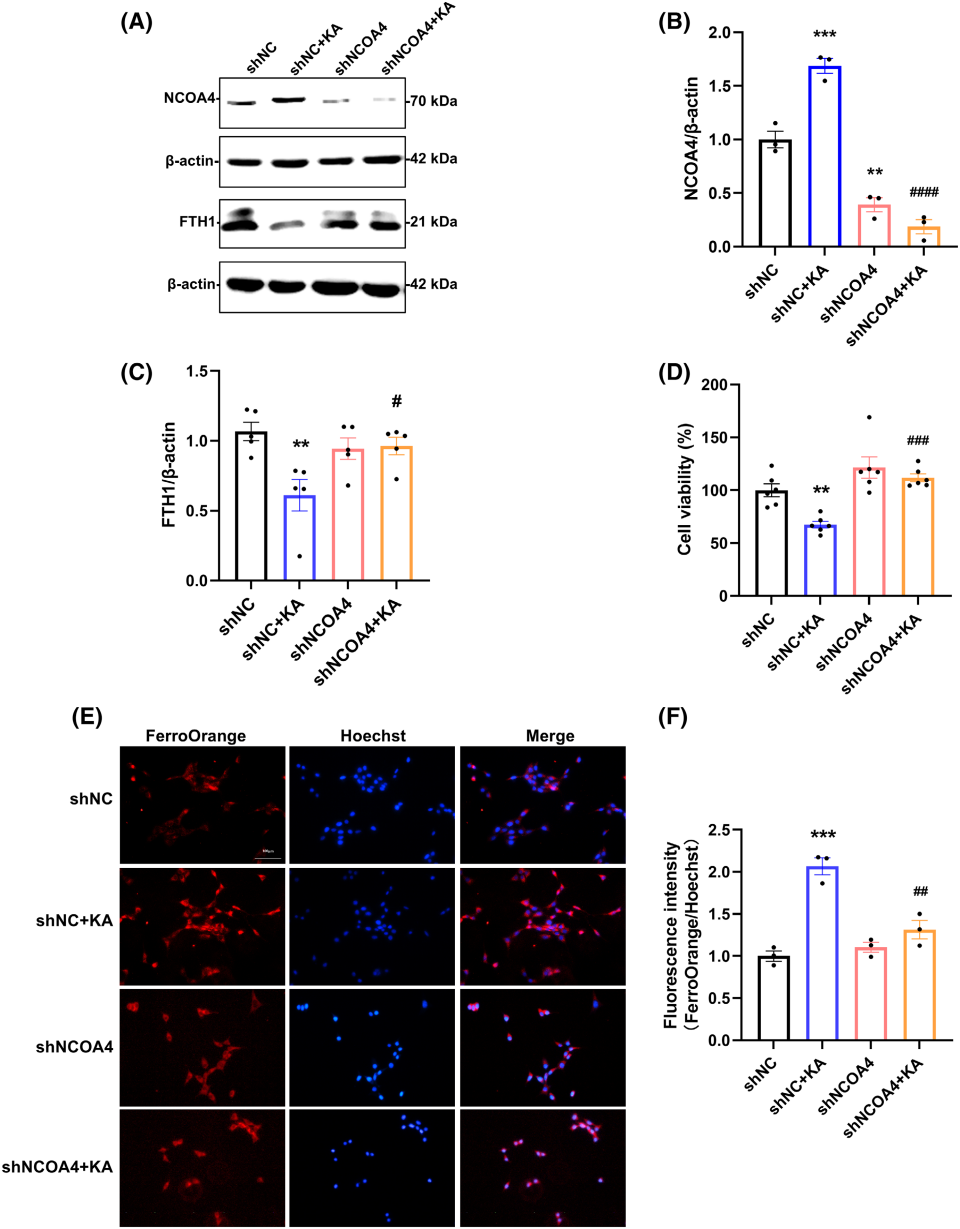
Knockdown of NCOA4 affects excitotoxicity neuronal activity, Fe^2+^ levels, and FTH1 protein expression level. (A–C) Representative bands and semiquantitation of western blots for detecting NCOA4 and FTH1 protein levels. (D) CCK‐8 detection of cell viability (*n* = 6). (E–F) FerroOrange fluorescence representative images (scale bar = 50 μm) and quantitative statistics for intracellular free Fe^2+^ (*n* = 3). Data are expressed as the mean ± SEM. **p < 0.01 vs. shNC group, ***p < 0.001 vs. shNC group, ^#^ *p* < 0.05 vs. shNC + KA group, ^##^ *p* < 0.01 vs. shNC + KA group, ^###^ *p* < 0.001 vs. shNC + KA group, ^####^ *p* < 0.0001 vs. shNC + KA group.

To explore whether NCOA4 could be a therapeutic target for neuronal excitotoxicity, it was necessary to establish stable cell lines. Since primary cortical neurons could not be used to establish stable cell lines, we used HT22 cell lines for the subsequent experiments. NCOA4 is needed for the induction of ferritinophagy. To investigate whether NCOA4 could serve as a therapeutic target for excitotoxicity, we transfected negative control shRNA and NCOA4 shRNA into HT22 cells and assessed interference efficiency using western blot. As shown in the results, the expression level of NCOA4 in shNCOA4 cells was decreased compared with shNC cells (Figure 5A,B). After KA treatment of shNC cells, the expression of FTH1 was downregulated. shNCOA4 could reverse KA‐induced FTH1 downregulation (Figure 5A,C). Furthermore, we investigated the protective effect of NCOA4 knockdown on excitotoxicity. NCOA4 knockdown cells exhibited better survival than shNC cells after KA treatment (Figure 5D). We detected the level of free Fe^2+^ in shNC and shNCOA4 cells using the FerroOrange probe and found that the Fe^2+^ level significantly increased in shNC cells after KA treatment, while the change in Fe^2+^ level in shNCOA4 cells was mild (Figure 5E,F). These results suggest that NCOA4 reduction can effectively combat oxidative stress and cell death caused by excitotoxicity‐induced increases in active iron levels.

## 4 DISCUSSION

Glutamate is the most common neurotransmitter in the CNS and the main excitatory amino acid. Overstimulation of its receptors can cause neuronal depolarization and an excessive influx of calcium ions, ultimately leading to neuronal death.^3^ It has been shown that excessive or prolonged exposure to excitatory amino acids can cause neuronal damage and death, which play a crucial role in the pathogenesis of neurodegeneration.^28^ Glutamate‐mediated excitotoxicity has been found to play an important role in the progression of neurodegenerative diseases such as AD, PD, and ALS.^29^, ^30^ In this study, an excitotoxicity model was established using an exogenous glutamate analogue, KA, both in vitro and in vivo, to explore potential therapeutic targets for neurodegenerative diseases.

The death of neurons is a core issue in excitotoxicity and neurodegenerative diseases. Interfering with specific programmed cell death processes is an important research strategy. Ferroptosis is a newly discovered form of programmed cell death that is characterized by the accumulation of a large amount of iron ions, lipid peroxidation and ROS accumulation, and abnormal mitochondrial morphology and has been reported to be related to nervous system dysfunction.^31^ In an early study, mitochondrial outer membrane rupture, crista shrinkage, iron imbalance, and lipid peroxidation were observed in KA model mice. Our research group also found an increase in the level of ACSL4 protein and a decrease in glutathione peroxidase 4 (GPX4) activity. Moreover, the occurrence of ferroptosis in the KA model was confirmed by using effective ferroptosis inhibitors.^32^ Therefore, we aimed to explore effective targets for inhibiting ferroptosis to rescue neuronal damage caused by KA.

An increase in free iron levels and iron deposition has been found in the brains of patients with various neurodegenerative diseases.^33^ Our research group found that in the excitotoxicity model induced in vivo, the total iron and Fe^2+^ levels in brain tissue were significantly increased from 12 h onwards. Iron chelators are the most direct and effective method for removing iron ions, and their efficacy is often unsatisfactory, as they have difficulty penetrating through the blood–brain barrier.^34^ Therefore, we attempted to explore the reasons for the increase in Fe^2+^ levels and found that the early changes in iron transmembrane transport‐related proteins were not significant in the KA model, while excessive degradation of ferritin was involved.

During the iron‐induced cell death cascade, the Fenton reaction activated by the abnormal increase in free Fe^2+^ is considered the “switch” that leads to a subsequent free radical chain reaction.^35^ We propose controlling Fe^2+^ in the KA model as a therapeutic strategy for rescuing neurons. In the in vitro model of KA, we demonstrated the efficacy of chelating iron ions with DFO in inhibiting iron‐induced cell death and rescuing neurons, providing favorable evidence for the feasibility of alleviating excitotoxicity by controlling the Fe^2+^ “switch”.

Autophagy is a cellular metabolic pathway of self‐digestion that plays an important role in regulating cell survival. There is ample evidence that KA can activate the autophagy/lysosome pathway, but the relationship between autophagy and ferroptosis in excitotoxicity is still unclear. Ferritinophagy is a selective autophagy that was proposed in 2014 that involves FTH1 being transported by NCOA4 to lysosomes for degradation and subsequently releasing a large amount of Fe^2+^ ^19^ Ferritinophagy has been considered a possible mechanism of ferroptosis.^36^ Currently, there are limited research reports on the connection between ferroptosis and neuronal death, but ferroptosis may be a potential target for excitotoxicity and other diseases. This study showed that KA drove lysosomes to release more Fe^2+^. After inhibiting the autophagy pathway with 3‐MA, the decrease in FTH1 levels caused by KA was restored to normal levels, and the upregulation of ferroptosis‐related indicators was also controlled, indicating that KA induces an increase in Fe^2+^ levels through autophagy, which then activates ferroptosis and causes neuronal damage.

NCOA4 was initially identified as a coactivator for multiple nuclear hormone receptors. It is primarily localized in the cytoplasm and affects the ligand binding specificity of androgen receptors. Moreover, NCOA4 has been associated with androgen‐independent prostate cancer.^37^ Recent studies have identified NCOA4 as a functional autophagic receptor through proteomics identification.^19^ Thus, we propose the hypothesis that NCOA4 can serve as a target for the treatment of excitotoxicity. In our research, an increase in NCOA4 protein expression levels was observed following KA treatment. Interfering with NCOA4 effectively increased cell viability and reduced Fe^2+^ levels, providing a simple strategy for the prevention or treatment of excitotoxicity by interfering with NCOA4. However, whether inhibiting NCOA4 can rescue lipid peroxidation and iron death induced by KA has not been fully proven, and we will continue to study the ferroptosis resistance of NCOA4 in excitotoxicity.

## 5 CONCLUSIONS

**FIGURE 6:**
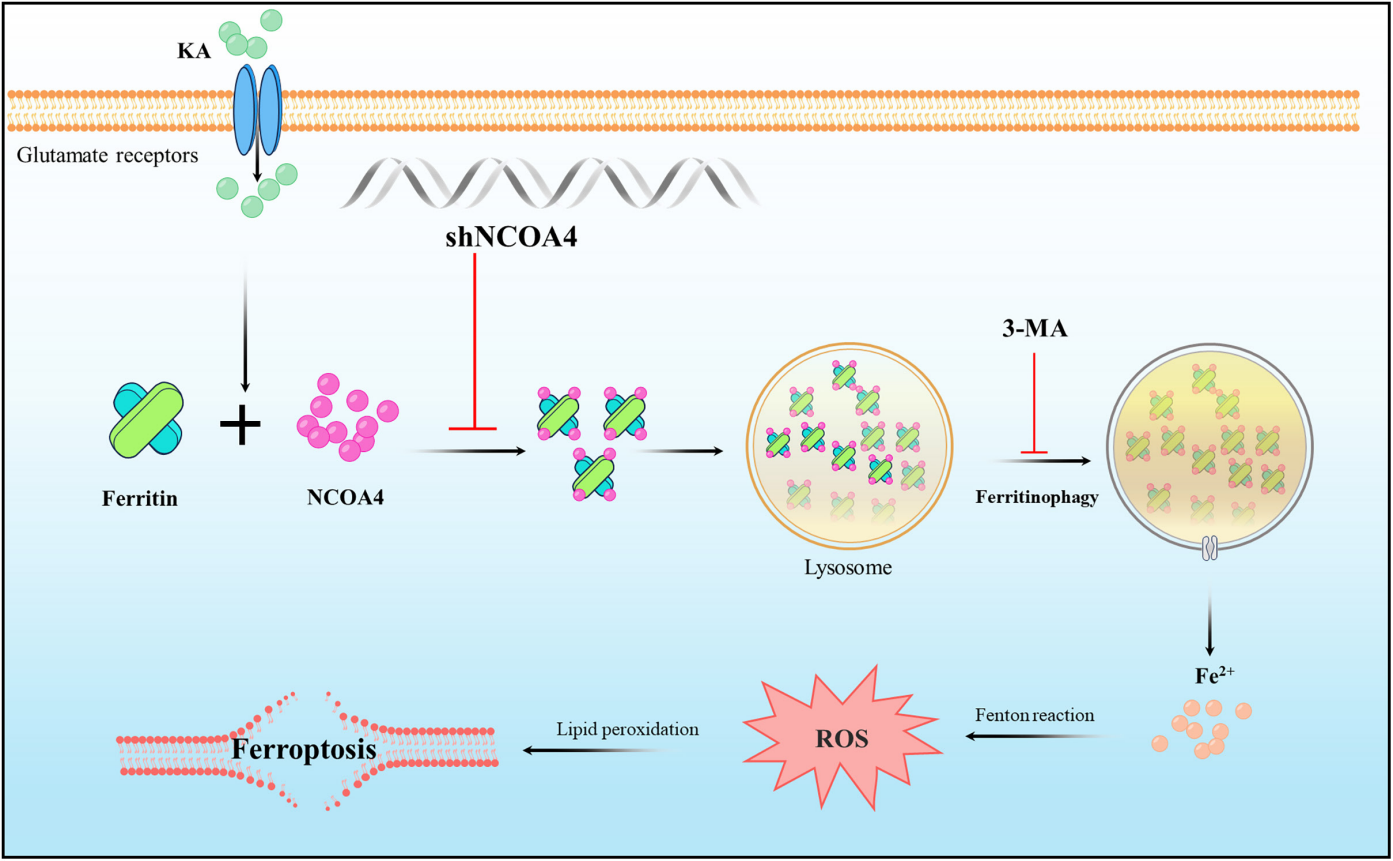
KA‐induced excitotoxicity leads to ferroptosis of neurons by activating ferritinophagy. 3‐MA effectively inhibited KA‐induced ferroptosis. KA activates ferritinophagy in neurons. Interference with NCOA4 effectively protects against neuronal damage caused by KA.

In conclusion, these results have shown that KA‐induced excitotoxicity is caused by the activation of NCOA4‐dependent ferritinophagy. This process leads to an increase in the release of reactive iron species, resulting in neuronal iron overload and ferroptosis, which ultimately causes damage to neurons. NCOA4, a regulatory target of ferritinophagy, has the potential to modulate neuronal resistance to excitotoxicity (Figure 6).

## AUTHOR CONTRIBUTIONS

Yan Wang and Yi‐Yue Jiang contributed to the conception of the study; Yi‐Yue Jiang and Wei‐Long Wu performed and edited the manuscript; Wei‐Long Wu, Jia‐Ni Huang, Na Liu, Jing Wang, and Xiao‐Rui Wan performed the data analyses; Yan Wang and Zheng‐Hong Qin conceived and supervised the manuscript.

## CONFLICT OF INTEREST STATEMENT

The authors have declared no conflict of interest.

## Supporting information


Data S1.

## Data Availability

The authors declare the availability of data and material.
