# Supplementary material for: KA‐mediated excitotoxicity induces neuronal ferroptosis through activation of ferritinophagy
Source: CNS Neurosci Ther. 2024 Sep 22;30(9):e70054. doi: 10.1111/cns.70054 (PMC11416743; doi:10.1111/cns.70054)

Supplemental Files

Full unedited blot for Figure 2A

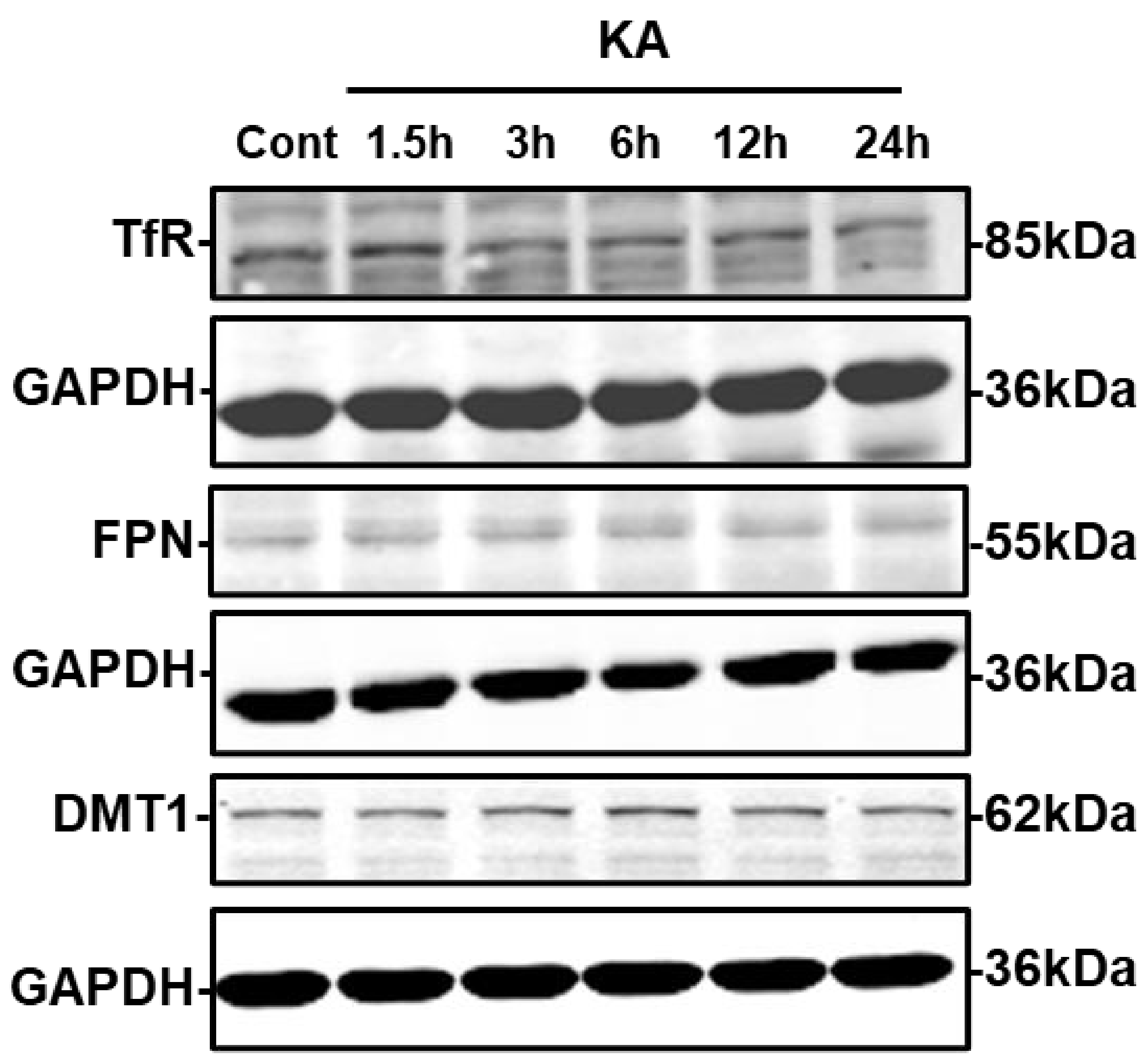

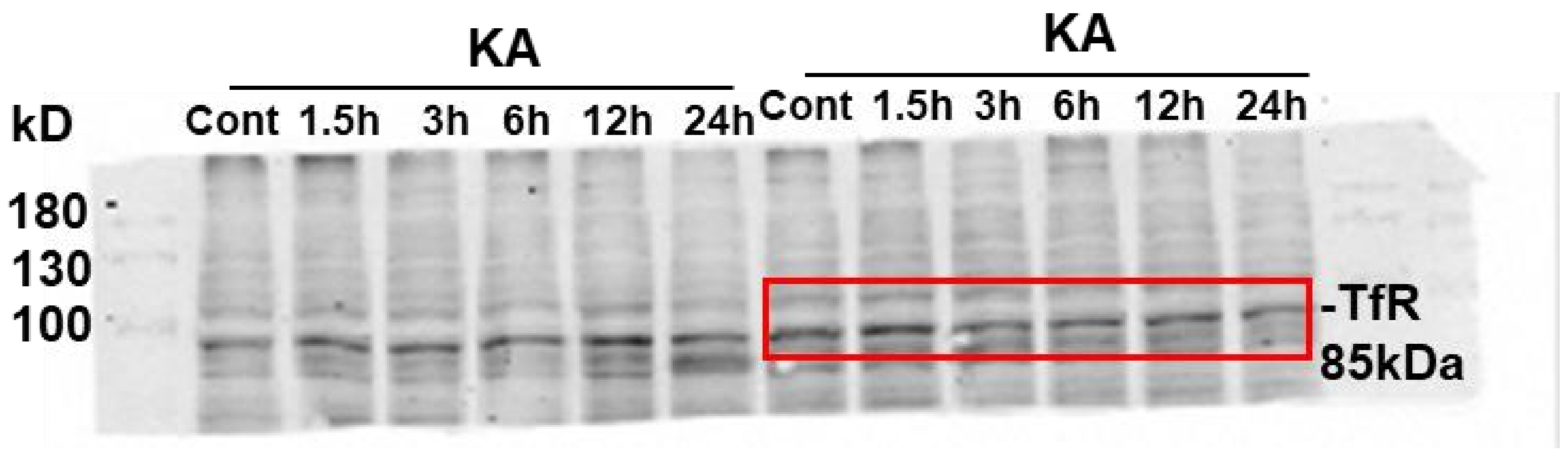

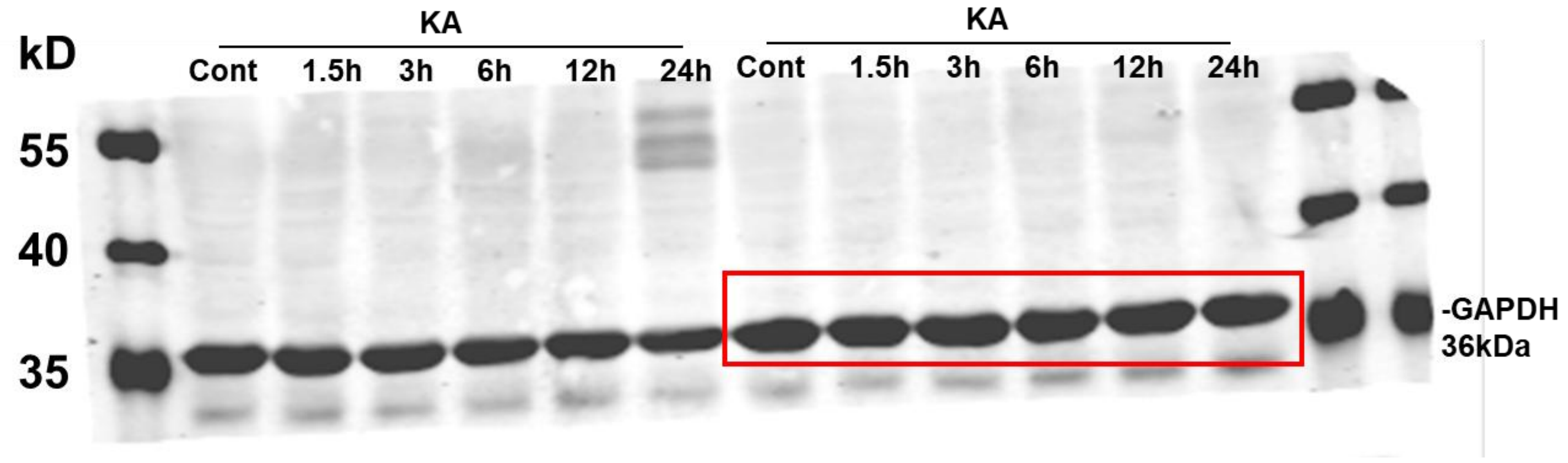

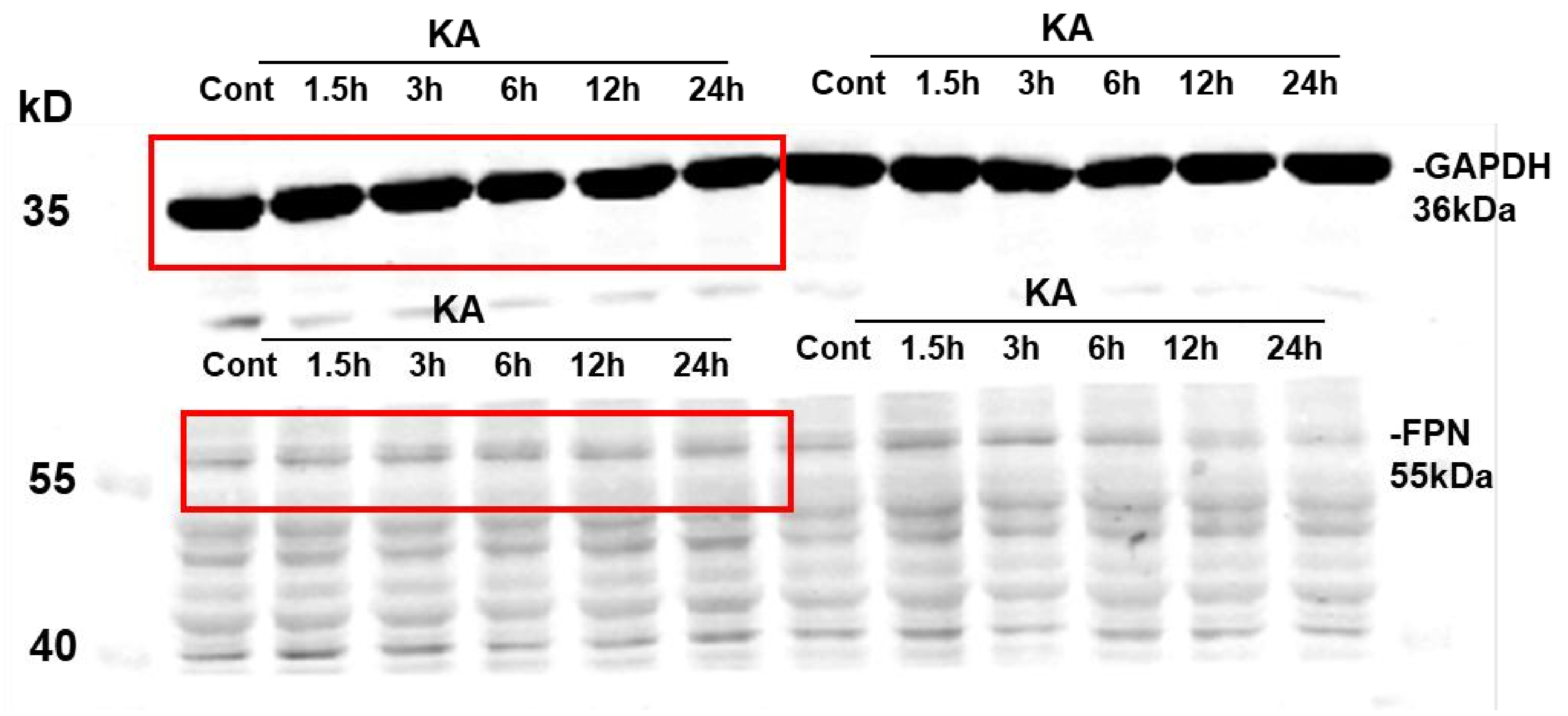

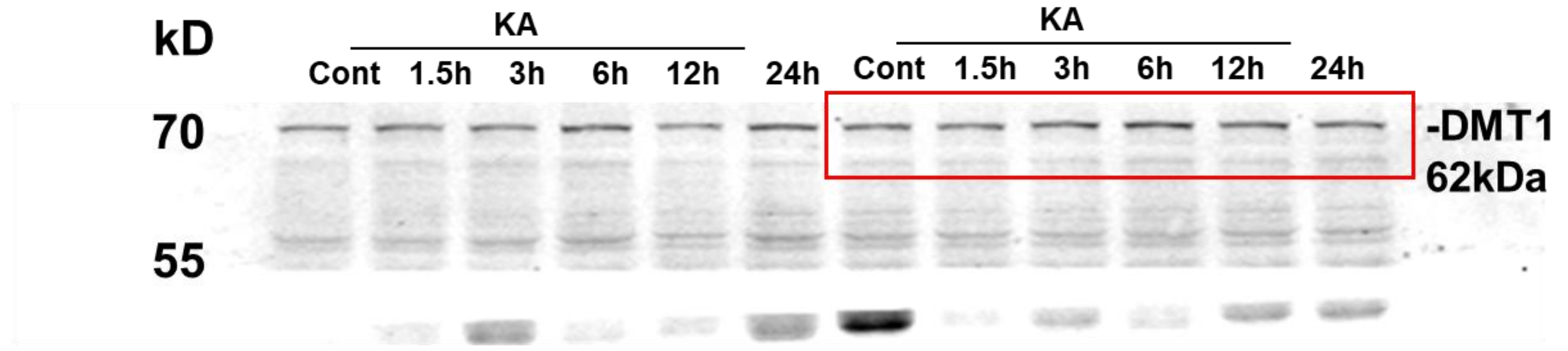

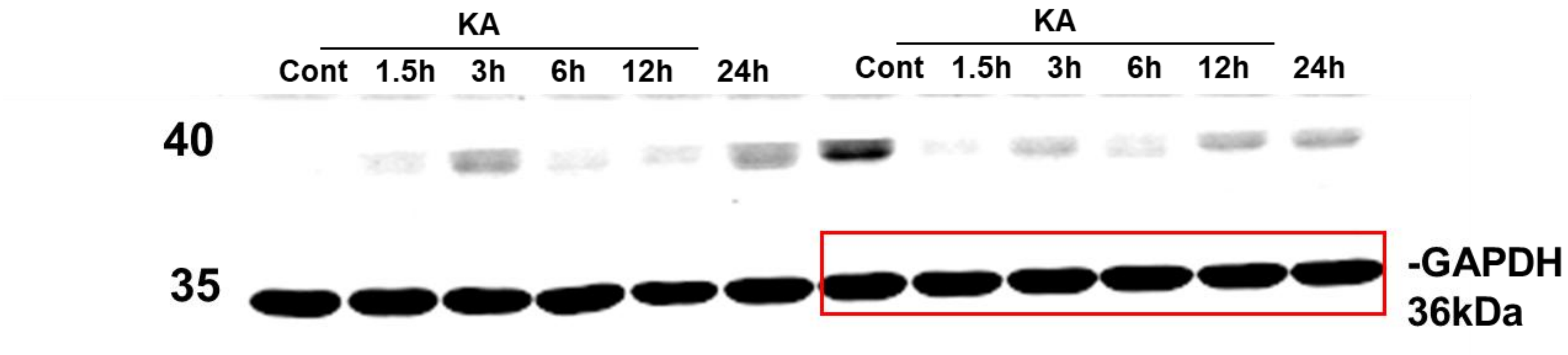

Full unedited blot for Figure 2E

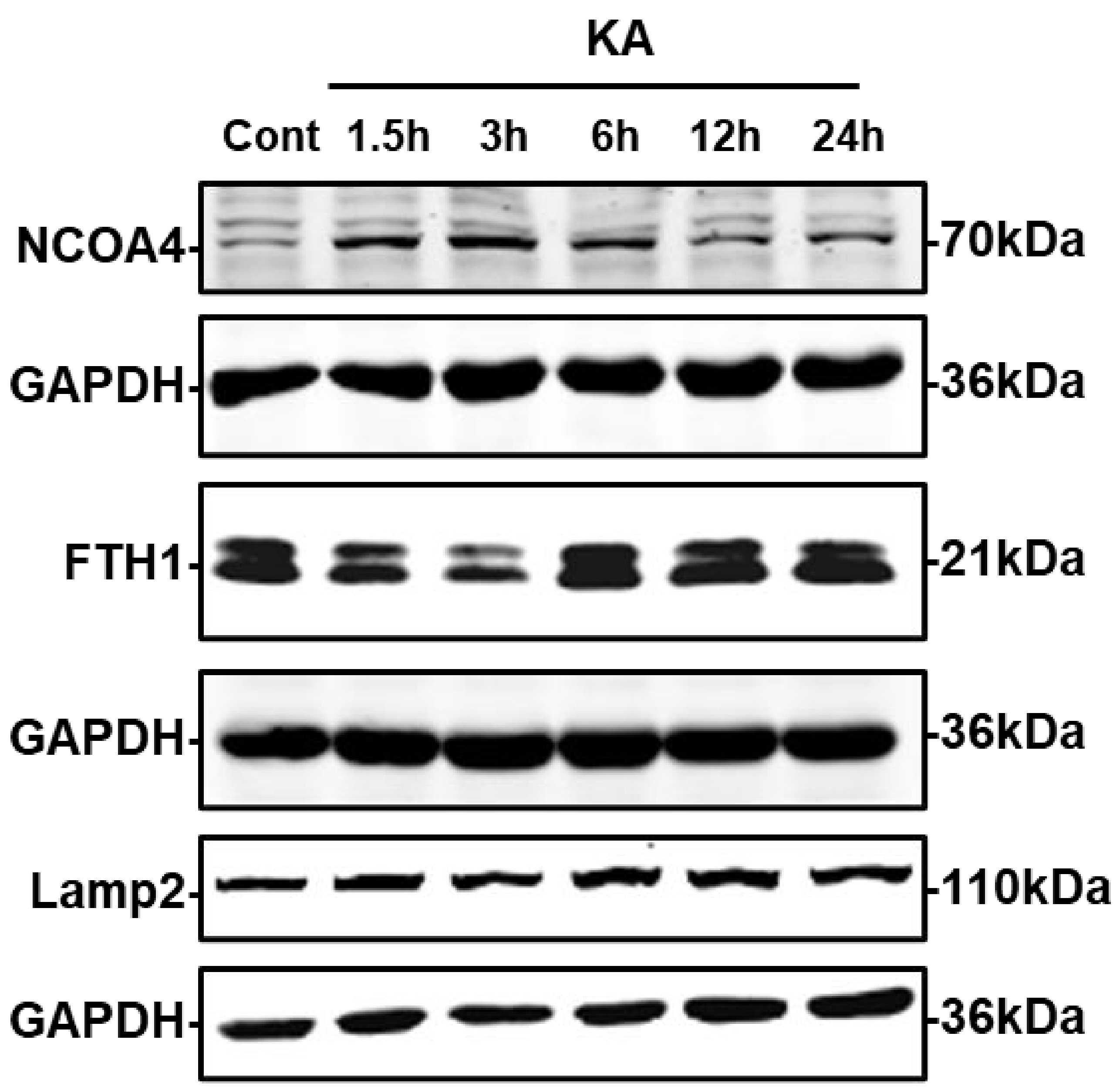

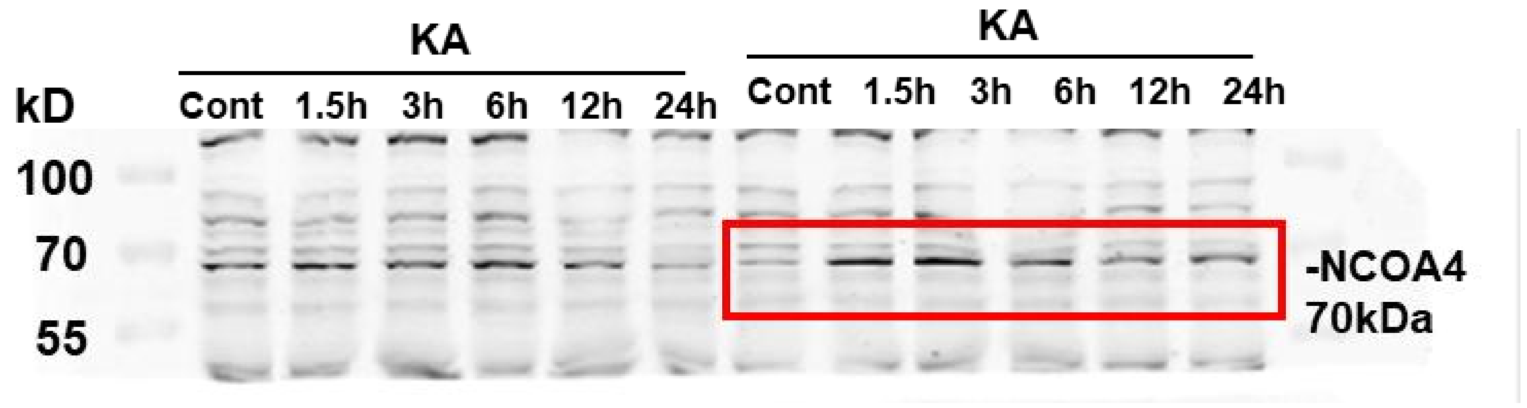

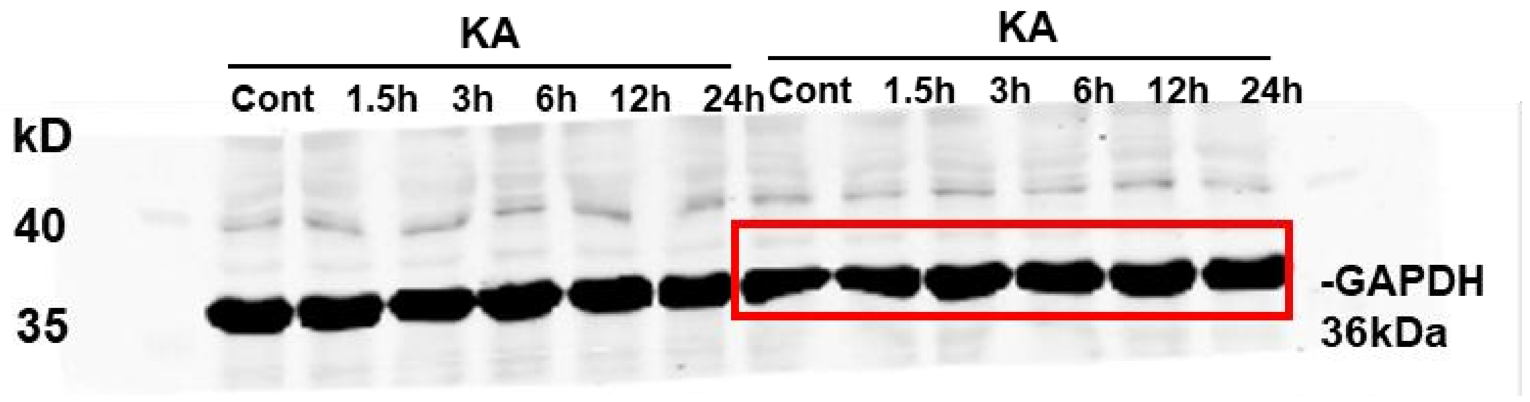

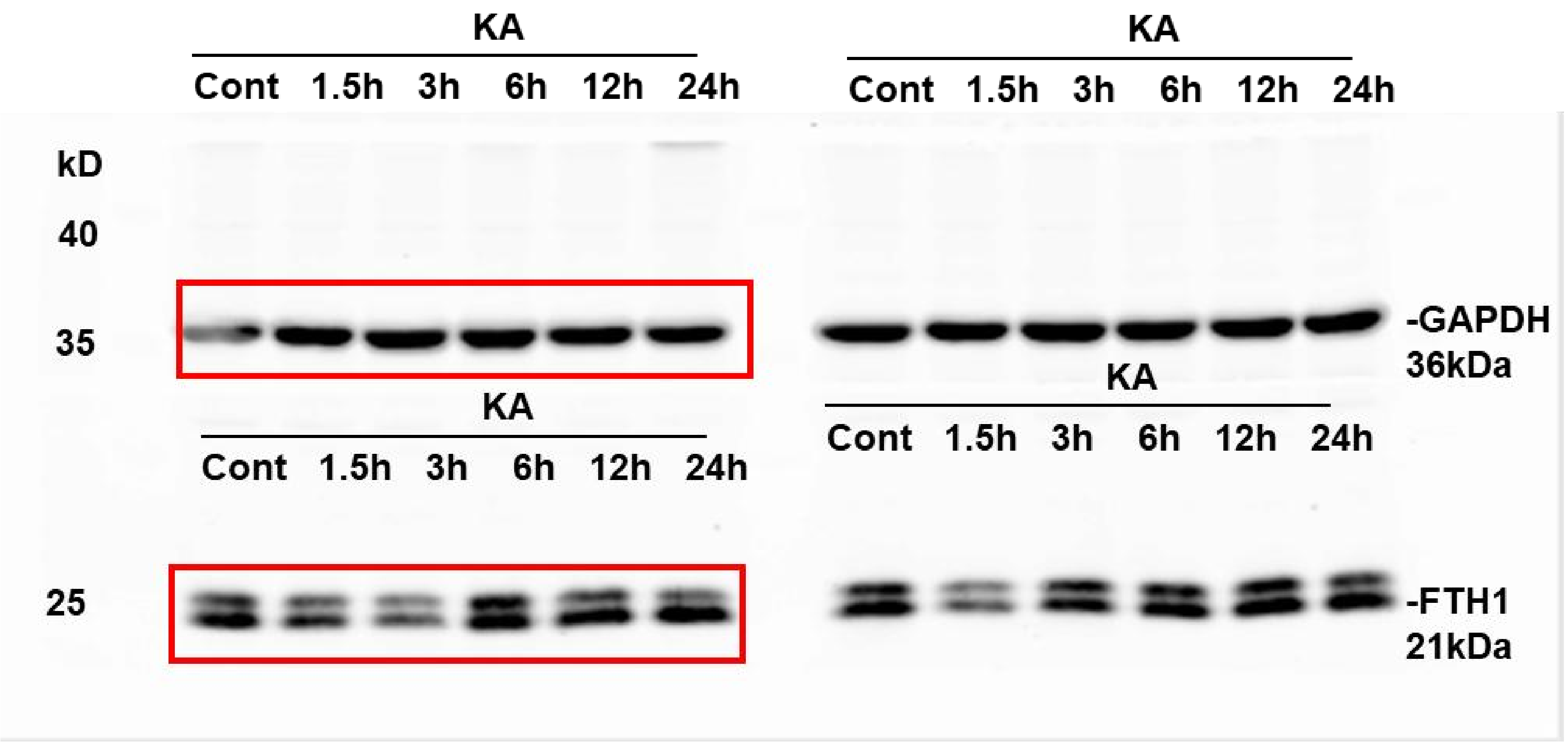

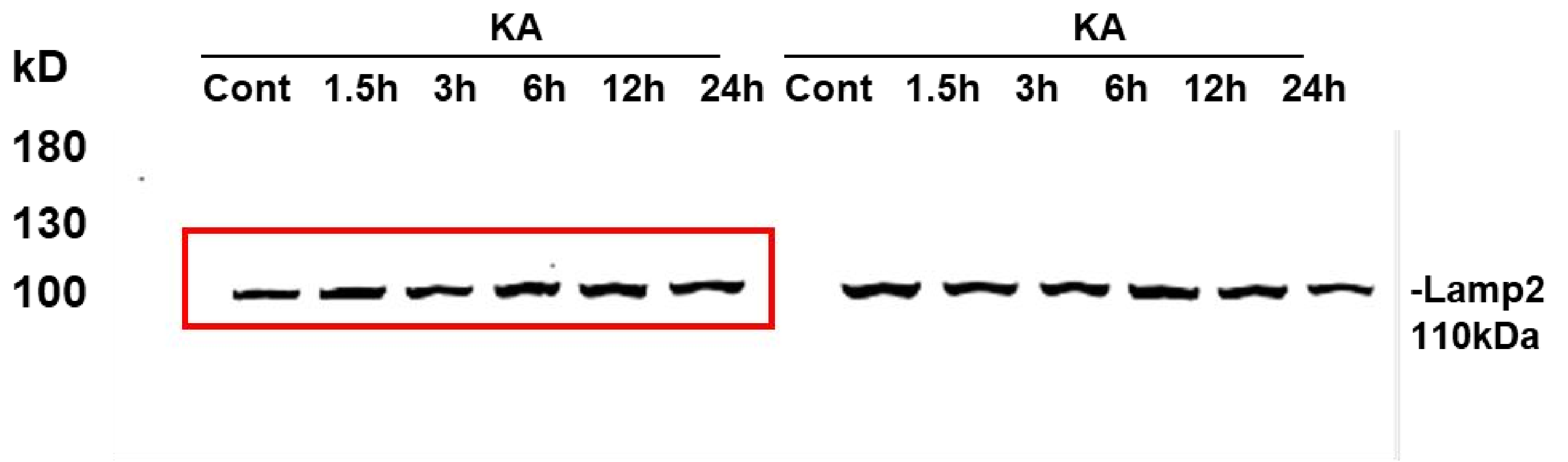

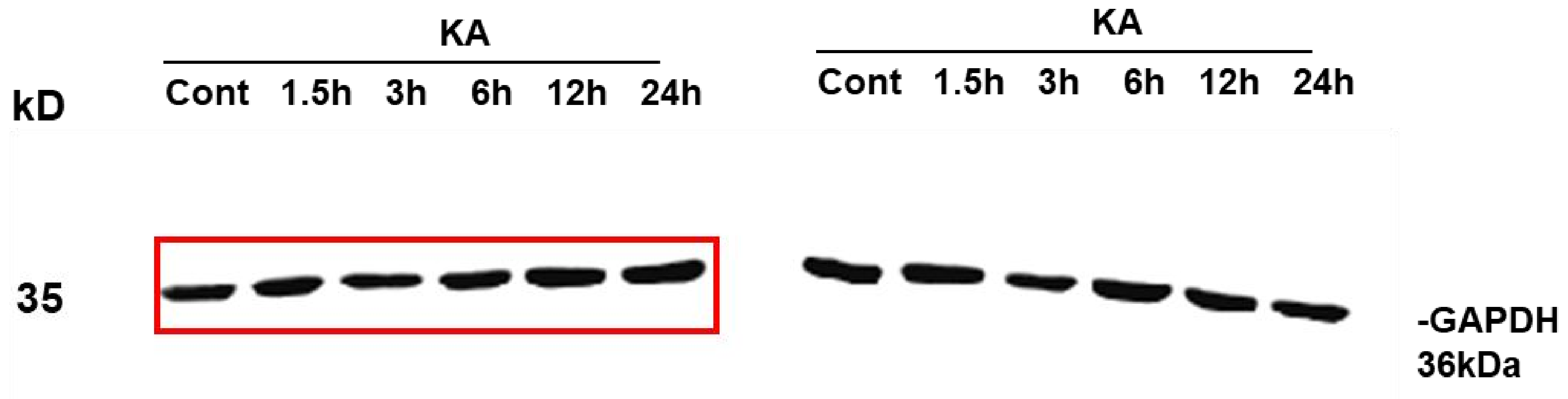

Full unedited blot for Figure 4A

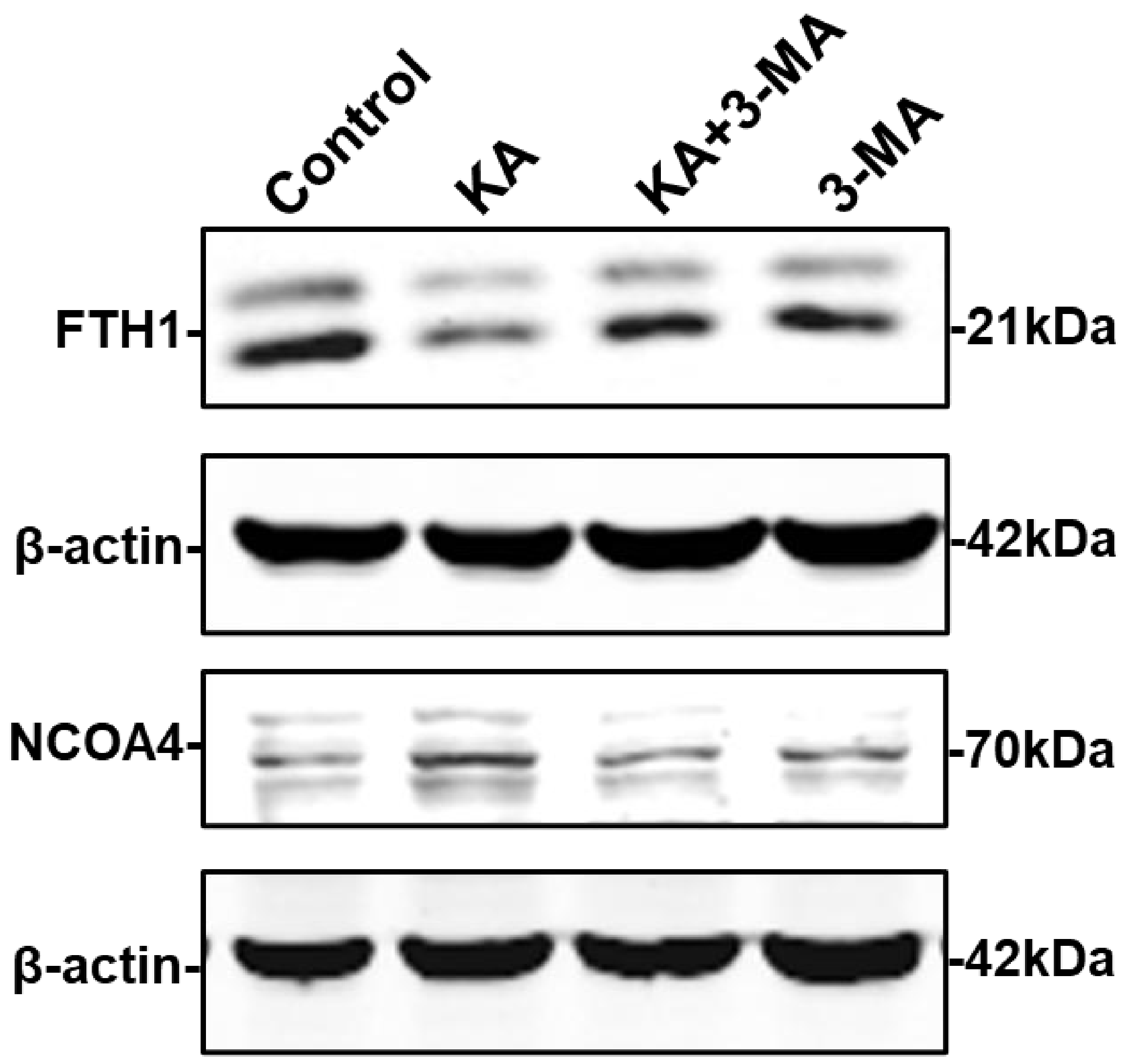

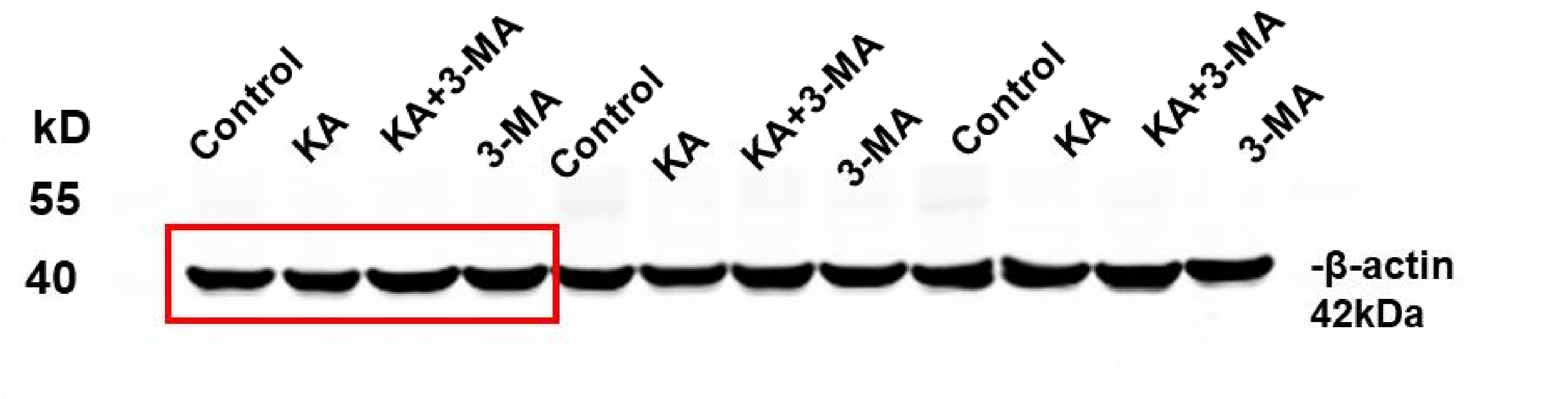

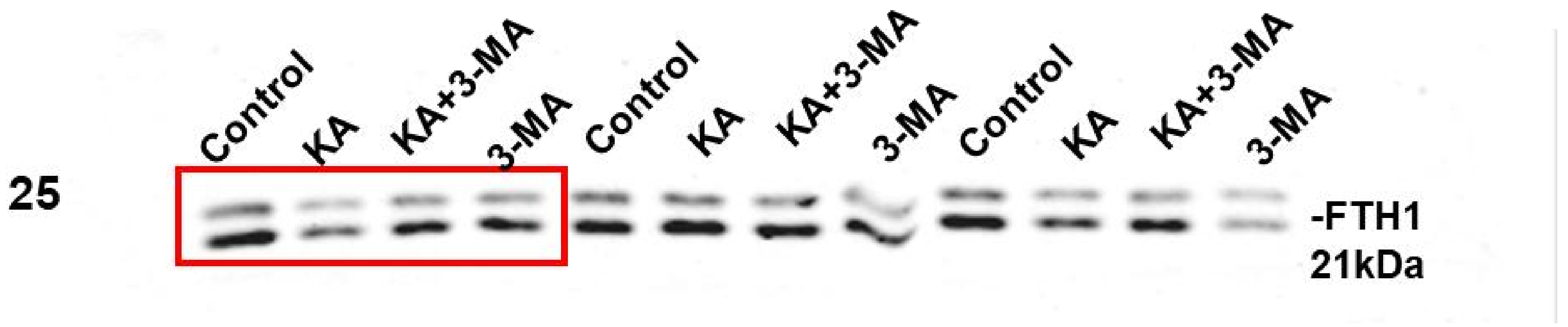

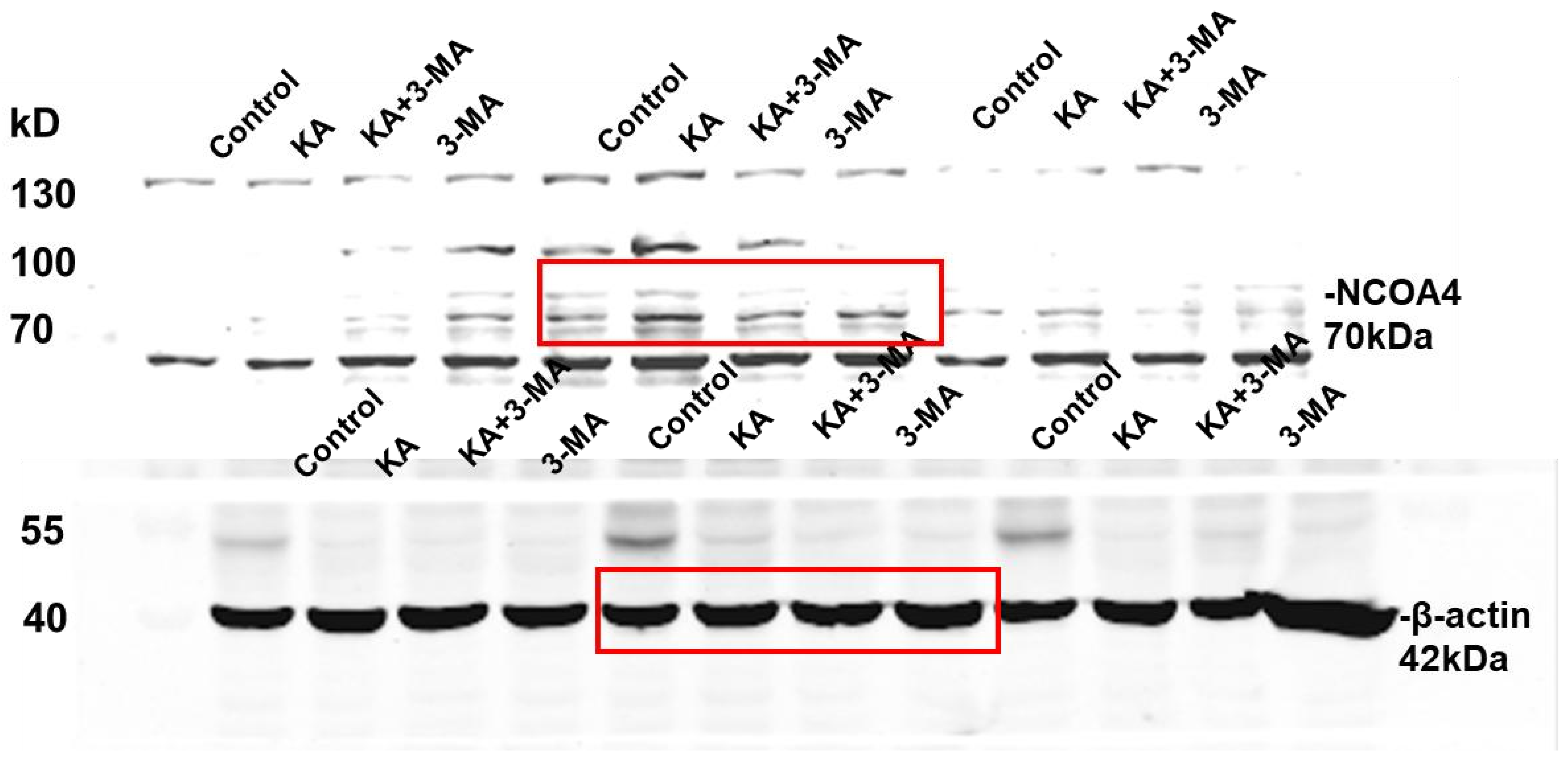

Full unedited blot for Figure 5A

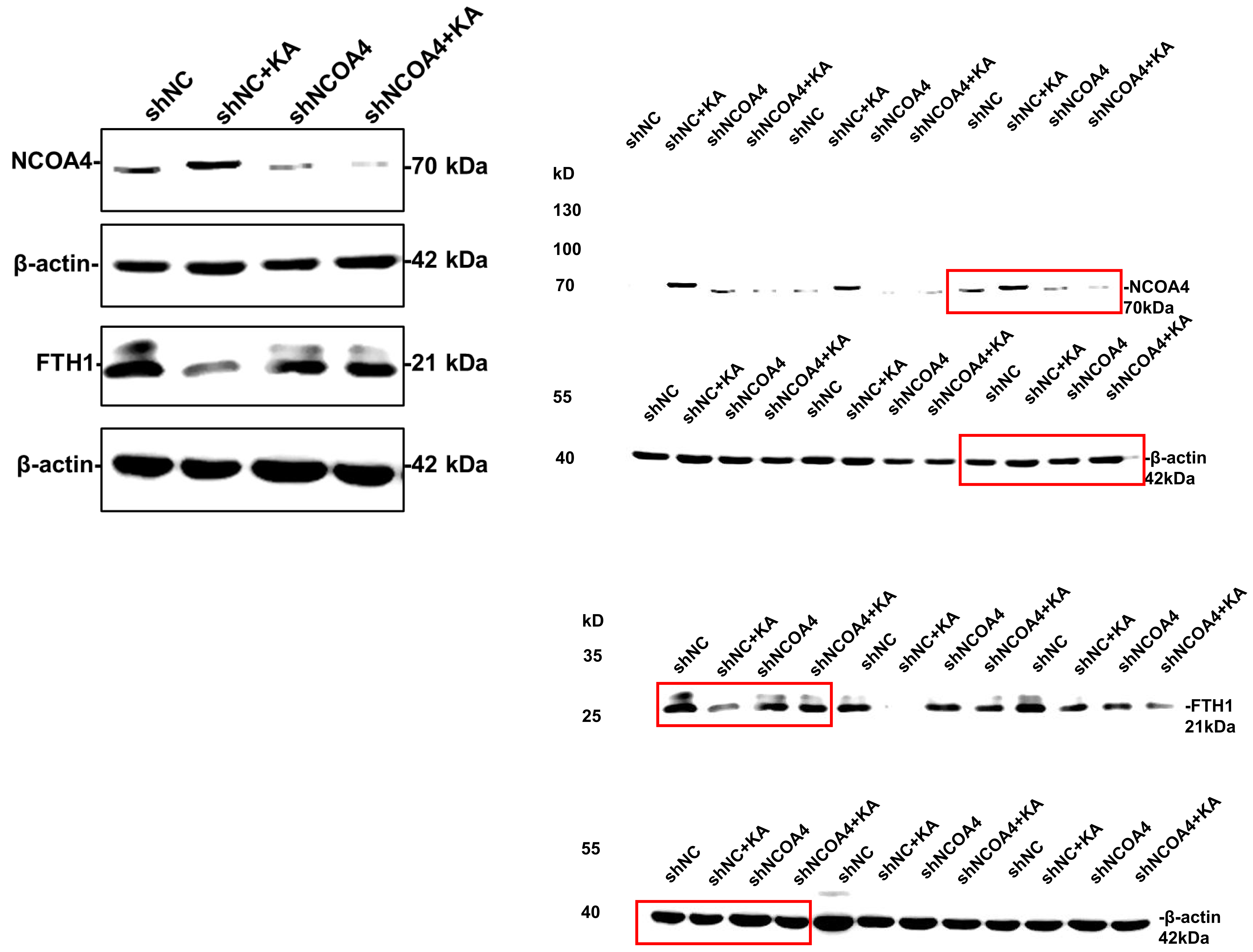

Supplement: Supplementary file 1 — Data S1. [file CNS-30-e70054-s001.pdf]
